# A novel study of Copy Number Variations in Hirschsprung disease using the Multiple Ligation-dependent Probe Amplification (MLPA) technique

**DOI:** 10.1186/1471-2350-10-119

**Published:** 2009-11-19

**Authors:** Rocío Núñez-Torres, Raquel M Fernández, Manuel López-Alonso, Guillermo Antiñolo, Salud Borrego

**Affiliations:** 1 Unidad de Gestión Clínica de Genética, Reproducción y Medicina Fetal, Hospital Universitario Virgen del Rocío, (Manuel Siurot s/n), Seville, (41013), Spain; 2 CIBER de Enfermedades Raras (CIBERER), (C/Álvaro de Bazán 10), Valencia, (46010), Spain; 3 Unidad de Gestión Clínica de Cirugía Infantil, Hospital Universitario Virgen del Rocío, (Manuel Siurot s/n), Seville, (41013), Spain

## Abstract

**Background:**

Hirschsprung disease (HSCR) is a congenital malformation of the hindgut produced by a disruption in neural crest cell migration during embryonic development. HSCR has a complex genetic etiology and mutations in several genes, mainly the *RET *proto-oncogene, have been related to the disease. There is a clear predominance of missense/nonsense mutations in these genes whereas copy number variations (CNVs) have been seldom described, probably due to the limitations of conventional techniques usually employed for mutational analysis.

**Methods:**

In this study we have aimed to analyze the presence of CNVs in some HSCR genes (RET, EDN3, GDNF and ZFHX1B) using the Multiple Ligation-dependent Probe Amplification (MLPA) approach.

**Results:**

Two alterations in the MLPA profiles of *RET *and *EDN3 *were detected, but a detailed inspection showed that the decrease in the corresponding dosages were due to point mutations affecting the hybridization probes regions.

**Conclusion:**

Our results indicate that CNVs of the gene coding regions analyzed here are not a common molecular cause of Hirschsprung disease. However, further studies are required to determine the presence of CNVs affecting non-coding regulatory regions, as well as other candidate genes.

## Background

Hirschsprung disease (HSCR, OMIM 142623) is a congenital malformation of the hindgut produced by a disruption in neural crest cell migration during embryonic development. This disorder results in an absence of intramural ganglion cells in the submucosal and myenteric plexuses, producing a functional intestinal obstruction [1,2]. HSCR has an estimated incidence of 1/5000 in live births, with a non-mendelian inheritance, reduced penetrance and male predominance. Although familial forms exist, the vast majority of cases are sporadic. In addition, HSCR can present as an isolated trait (70% of the cases), or in association with chromosomal abnormalities, neurodevelopmental disorders and a variety of additional isolated anomalies and syndromes [2].

HSCR has a multifactorial genetic etiology with several genes described as being associated with isolated or syndromic forms of the disease. These genes are usually involved in the neural crest cell development and migration that gives rise to the enteric nervous system (ENS). Undoubtedly, the *RET *proto-oncogene is considered the major disease-causing locus in HSCR and has been extensively studied in different series of HSCR patients. Traditional mutations within *RET *coding sequence have been detected in up to 50% of familial cases and in 10-20% of sporadic forms of the disease [1,2]. Moreover, a common non-coding *RET *variant within a conserved enhancer-like sequence in intron 1 has been reported to be significantly associated with HSCR susceptibility and to make a 20-fold greater contribution to risk than conventional coding mutations do [3]. HSCR can, therefore, be defined as a complex disorder with non-mendelian inheritance that requires *RET *and other interacting disease susceptibility alleles. As is the case with many other complex diseases, the manifestation of the phenotype may result from the combination of pathogenic events in one or several genes, acting in an additive or multiplicative manner. These additional events are therefore necessary not only to explain the disease incidence but also its complex pattern of inheritance.

Mutations in other genes encoding proteins involved in the RET pathway, such as *GDNF, NTN*, or *GFRA1*, have been also reported in HSCR patients. However, they affect a minority of cases and frequently appear in combination with additional contributory factors, like *RET *mutations or trisomy 21 [1,2].

The second major HSCR gene is the one encoding the Endothelin receptor B (*EDNRB*), which presents a mutational rate of approximately 5% in HSCR patients. Also participating in the same EDNRB pathway, *EDN3 *and *ECE1 *have seldom been reported to present mutations related to syndromic forms of HSCR [1,2].

A third pathway involved in the ENS formation, NTF3-NTRK3, showed evidence of being related to HSCR, as mutations in both *NTF3 *and *NTRK3 *genes were identified in isolated patients [4,5]. Nevertheless, additional studies are required to determine their real involvement in the disease.

Finally, other genes encoding for transcription factors (*SOX10, ZFHX1B*, *PHOX2B, TCF4*), or other cell elements (*KIAA1279*), have been eventually related to different syndromes that include Hirschsprung [1,2].

Regarding the nature of the mutational events leading to HSCR phenotype, there is a clear predominance of missense/nonsense mutations, especially in *RET *and *EDNRB *http://www.hgmd.cf.ac.uk/ac/index.php. Small deletions or insertions and splicing mutations in *RET *have been found in a few patients (see HGMD), whilst they are rarely reported in other genes. In contrast, no duplications and only a gross deletion affecting the entire sequence of *RET *have been reported to date [6]. A possible explanation for this low rate of copy number variations (CNV) related to HSCR is that the techniques usually employed for mutational screening, such as SSCP, dHPLC or sequencing analysis, are not adequate to identify these types of rearrangements.

In light of this information, in the present study we have sought to study the presence of CNVs in *RET*, the "major HSCR gene" in our series of patients, using the Multiple Ligation-dependent Probe Amplification (MLPA) technique, which is one of the best methods for detecting alterations in genes dosages [7]. In addition, another 3 genes responsible for a minority of the cases, (*GDNF, EDN3, ZFHX1B*) were simultaneously analyzed as the MLPA kit used also contained probes for their detection.

## Methods

### Patients and Control Subjects

A total of 208 isolated HSCR patients from Spain (22% female, 77% male) were involved in this study. 188 of these patients were sporadic cases, whilst 20 were familial forms belonging to 13 different families. *RET *coding mutations had been found in 23 of the 188 sporadic cases (mutational rate: 12%), and in 4 of the 13 familial forms (mutational rate: 31%) [reference [8] and unpublished data]. In addition, 80% of the patients were heterozygous or homozygous for the "*RET *enhancer mutation" [reference [9] and unpublished data]. Regarding the mutational status for the remaining "HSCR genes", a multiplex family with 2 affected members presenting mutations of *RET*, *NTRK3 *and *EDN3 *genes [5,10], and a sporadic patient carrying a *NTF3 *mutation [4] are worthy of mention. Finally, *EDNRB *coding mutations were found in 5 sporadic patients and in 1 familial case (unpublished data). No other mutational events related to HSCR were found in the remaining HSCR genes analyzed by our group. Given the complex nature of Hirschsprung disease, in which the contribution of several loci seems to be necessary for the manifestation of the phenotype, we included our whole series of patients in the present study despite their mutational status for *RET *and other genes.

In addition, we also included a group of 10 normal controls comprising unselected, unrelated, race, age, and sex-matched individuals. They were all healthy voluntary donors, who came to the Hospital for other reasons and did not present any symptoms suggestive of HSCR.

Informed consent was obtained from all the participants for clinical and molecular genetic studies. The study was approved by the Ethics Committee for clinical research in the Hospital Universitario Virgen del Rocio of Seville, and complies with the tenets of the declaration of Helsinki.

### MLPA analysis

The MLPA-Hirschsprung test kit (P169) was supplied by MRC Holland, Amsterdam, Netherlands. This kit contains 41 different probes designed to detect alterations in the copy number of one or more exons in four genes involved in either isolated or syndromic forms of HSCR (*RET*, *ZFHX1B*, *EDN3 *and *GDNF*). In addition, the kit also contains 5 control fragments which hybridize with different regions of the genome. The MLPA reaction was performed in a MJ Research Thermal Cycler with a heated lid following MRC Holland DNA - detection quantification protocol http://www.mrc-holland.com/WebForms/WebFormMain.aspx?Tag=wl2zCji\rCGANQgZPuTixtCplCA1mmwJoFo/xHPnTgc|.

Fragment analysis was performed using the 3730 DNA analyzer (Applied Biosystems, Foster City, CA, USA) and for data analysis we used GeneMarker v 1.6 (Softgenetics L.L.C). We normalized the samples by using the peak heights values, and included control individuals who had previously been confirmed to have no CNV of the studied genes. Furthermore, duplicate assays were performed to check the accuracy of the analysis results.

## Results

**Figure 1:**
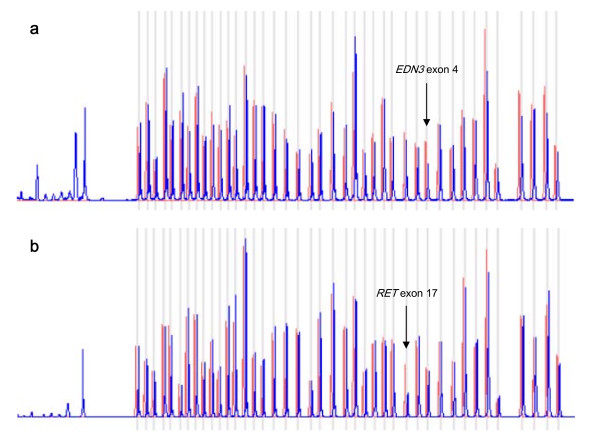
**MLPA profiles for control individuals (red), and for the HSCR patients (blue)**. For these HSCR cases, an increase in the dosage of *EDN3 *exon 4 (a) and *RET *exon 17 (b) can be observed.

The main aim of this study was to determine if our HSCR patients were carriers of variations of the number of copies of *RET *and other 3 genes related to the disease. It has been previously described that polymorphisms or single base mutations located within the probe binding regions may affect MLPA results [7]. Therefore, we carried out an screening of point mutations prior to the MLPA tests, by denaturing high performance liquid chromatography (dHPLC) and direct sequencing of the coding regions of the 4 genes included in the kit. As a result, several point sequence changes were identified, the majority of them being common polymorphisms[8,10,11]. Subsequently, we performed the MLPA technique on our series of 208 patients with the MLPA-Hirschsprung test kit (MRC Holland) analyzing *RET *(exons 1-21), *GDNF *(exons1-4), *EDN3 *(exons 1-5) and *ZFHX1B *(exons 1-10). Our analysis revealed that two of these patients presented an apparent alteration in the gene dosage of *RET *and *EDN3 *respectively, that consisted in a signal decrease of 50% when compared with the control individuals. The profiles obtained for both individuals are shown in Figure 1. The rest of the patients presented normal dosages for all the exons studied.

**Figure 2:**
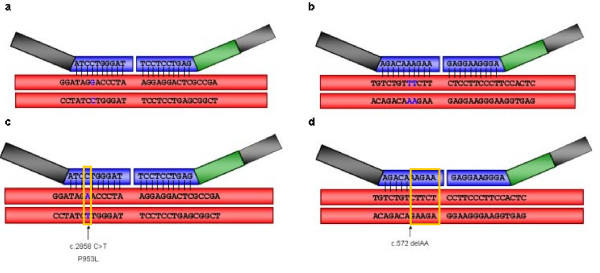
**Schematic representation of the hybridization probe regions of *RET *exon 17 (a) and *EDN3 *exon 4 (b)**. The specific positions where mutations affect the hybridization processes are shown in c and d.

We therefore investigated the exact probe binding sites for the 2 altered exons and sought to confirm if they were affected by the presence of any sequence variant previously detected in the corresponding patients. The first patient presented a decrease in exon 17 of *RET*, but the neighboring exons' dosages were completely normal. The previous *RET *mutational screening by dHPLC of this patient had shown that he carried the point mutation c.2859T>A (p.Pro953Lys) in heterozygosis, which as suspected is located within the hybridization probe region corresponding to exon 17 (unpublished data, Figure 2). The second patient presented a decrease in the dosage of exon 4 of *EDN3 *and an inspection of the previous results from the *EDN3 *dHPLC screening showed that he carried a 2 nucleotides deletion, c.572delAA (p.Lys191ArgfsX59), affecting the corresponding hybridization probe region ([11], Figure 2). Both *RET *c.2859T>A and *EDN3 *c.572delAA variants were found to be absent in 200 normal controls [8,10,11], and *in silico *analysis predicted a damaging effect for both of them, which suggest that these variants may be involved in the manifestation of the HSCR phenotype in these patients, although probably in combination with still unidentified genetic events at other loci [8,10,11].

## Discussion

HSCR has a complex genetic etiology and several genes have been reported to be implicated in the disease. The *RET *proto-oncogene has been described as the major HSCR gene, and over 100 mutations have been identified including large chromosomal deletions which encompass the whole *RET *sequence, microdeletions and insertions, splicing, and nonsense and missense mutations [2], the last mutations being the most frequent alterations found in HSCR patients. Missense/nonsense variants are also the most commonly described events in the remaining genes related to HSCR, in contrast with gross deletions or CNVs that have seldom been reported. Possible factors contributing to this fact could be that point mutations and small deletions/insertions are easily detected by typical screening methods based on conventional PCR, and large chromosomal rearrangements are identified by cytogenetic techniques, whereas none of the 2 approaches are powerful enough and adequate to detect CNVs affecting specific regions corresponding to a few kilobases [7]. Therefore we decided to analyze our HSCR series using the MLPA technique, which allows medium-size alterations in the number of copies to be detected, by simultaneously screening *RET*, *EDN3*, *GDNF *and *ZFHX1B*.

2 patients with alterations in the MLPA profile of exon 17 of *RET*, and exon 4 of *EDN3 *respectively were identified. However, a detailed inspection showed that such variations were not actual changes of the copy number but a fail on the corresponding probes hybridization processes. MLPA is a comparative method designed for the effective detection of variations in gene dosage making estimating the gene copy number possible. This method is being used extensively in the molecular diagnosis of a wide range of diseases caused by deletions or duplications of one or more exons in specific genes. The main disadvantage of using this technique is the short length of the hybridizing region of the MLPA probes (20-30 nucleotides), which means that nucleotide mismatches at the corresponding probe binding sites may hinder the hybridization process and lead to an apparent deletion being detected, as has been observed here. Although other techniques such as Comparative Genomic Hibridization arrays (CGH array) are considered the best method for CNVs detection, MLPA provides us with a way of identifying CNVs in a quick and simple way, that is effective enough to achieve our specific aims. Our results appear to indicate that CNVs are not a common molecular cause of Hirschsprung disease. In fact, of the 4 simultaneously analyzed genes, only *RET *has shown to have a major impact on the disease, while the rest are only responsible for a minority of cases. A possibility still remains that other HSCR genes that have not been evaluated in the present study may present CNVs responsible for a percentage of HSCR cases.

## Conclusion

Our results appear to indicate that CNVs in the coding region of *RET *and the other 3 HSCR related genes here tested are not a common molecular cause of Hirschsprung disease, at least in the Spanish population. It would be interesting to analyze other genes such as *EDNRB*, the second major gene in HSCR, in future MLPA studies. In addition, the MLPA probes used here have been designed for only scanning the coding sequence of these genes, meaning that intronic and UTR regions are missed out. Given the prominent role of the *RET *enhancer mutation within intron 1 in the context of HSCR (3), revealing the great relevance of the non-coding elements in the pathogenesis of the disease, it would be also advisable to include such regions in additional future MLPA analyses. Therefore, further studies are warranted to completely rule out CNVs as the molecular cause of some forms of Hirschsprung disease.

## Competing interests

The authors declare that they have no competing interests.

## Authors' contributions

RN-T and RMF carried out the molecular genetic studies, participated in the MLPA analysis and drafted the manuscript. ML-A recruited the HSCR patients and participated in the design of the study. SB and GA participated in the coordination of the study and helped to draft the manuscript. All authors have read and approved the final manuscript.

## Pre-publication history

The pre-publication history for this paper can be accessed here:

http://www.biomedcentral.com/1471-2350/10/119/prepub
